# Venus Flytrap (*Dionaea muscipula* Solander ex Ellis) Contains Powerful Compounds that Prevent and Cure Cancer

**DOI:** 10.3389/fonc.2013.00202

**Published:** 2013-08-20

**Authors:** François Gaascht, Mario Dicato, Marc Diederich

**Affiliations:** ^1^Laboratory for Molecular and Cellular Biology of Cancer (LBMCC), Hôpital Kirchberg, Luxembourg, Luxembourg; ^2^Department of Pharmacy, College of Pharmacy, Seoul National University, Seoul, South Korea

**Keywords:** chemoprevention, therapy, natural compound, *Dionaea*

## Abstract

Chemoprevention uses natural or synthetic molecules without toxic effects to prevent and/or block emergence and development of diseases including cancer. Many of these natural molecules modulate mitogenic signals involved in cell survival, apoptosis, cell cycle regulation, angiogenesis, or on processes involved in the development of metastases occur naturally, especially in fruits and vegetables bur also in non-comestible plants. Carnivorous plants including the Venus flytrap (*Dionaea muscipula* Solander ex Ellis) are much less investigated, but appear to contain a wealth of potent bioactive secondary metabolites. Aim of this review is to give insight into molecular mechanisms triggered by compounds isolated from these interesting plants with either therapeutic or chemopreventive potential.

## Introduction

Natural products derived from plants, animals, and microorganisms have traditionally been the main source of active medicinal compounds without a deep understanding of their mechanism of action. Emergence of resistance in different already known pathologies (cancer, bacterial infections) (1, 2), but also the emergence of other, yet incurable diseases (Alzheimer’s disease, Parkinson’s disease, AIDS …) (3), call for the discovery of novel therapeutic compounds and the improvement of efficiency of already known molecules (4, 5).

The term “secondary metabolites” refers to molecules, which are not directly involved in essential processes like development, growth, and reproduction compared to the primary metabolism. Synthesized by all living kingdoms (Archae, Bacteria, Protisae, Plantae, Fungi, and Animalia), these non-essential metabolites are different depending on the species and are classified according to their method of synthesis. Structurally highly diversified and complex and present in very small quantities, secondary metabolites account for often less than 1% of the total mass of organic carbon in the organism. Their level of synthesis can also depend on the physiological and the developmental stage of the organism but also environmental factors like the soil, climate, or weather. Synthesis of secondary metabolites can be induced after stimulation by stressors from diverse origins. Originally isolated from plants, recent researches have shown that some secondary metabolites are synthesized by symbiotic organisms like bacteria and not by the host organisms themselves and that other have symbiotic origins. The role of secondary metabolites is to ensure the survival of the organism in its environment. Some allow organisms to protect themselves against predators or herbivores, insects, pathogens but also to kill preys like snake and arthropod venoms or against other organisms for access to resources (light, water, and nutrients). Other metabolites can help to resist environmental stress (drought, nutrient deficiencies), attract pollinating insects (by color and odor), or to ensure symbiosis with other organisms. To date, more than 200,000 different secondary metabolites have been discovered and described. Some have been diverted from their original use by human and are now used in commercial preparations such as dyes, drugs, or insecticides (6–12).

### “Non-food” plants are also an attractive source of molecules with potential chemopreventive interest

Various chemopreventive and therapeutic compounds have been isolated from food plants. We can notably mention, flavonoids (carrots) including chalcones, isothiocyanates (cabbage), lycopene (tomatoes), indoles, organosulfides (garlic), and polyphenols (curcumin) (13–19).

Many compounds can also be found in food preparations made with fruits or vegetables like resveratrol from red wine (20–22) or catechins and procyanidins and polyphenols from cocoa (23, 24) or quercetin and kaempferol from honey (25).

However, many “non-food” plants from all around the World are also attractive sources for molecules with potential chemopreventive interests (26–29).

The underwater world is also rich in bioactive molecules with chemopreventive and anti-tumor potential. Among these secondary metabolites discovered in animal, fungi, micro-organism, or marine plants we can mention, for example heteronemin and hemiasterlin (sponges), kahalalide F. (sea slug), naphthopyrones (echinoderm), didemnin B (tunicate), and amphidinolides (algae) (12, 18, 30–34).

## The Venus Flytrap (*Dionaea muscipula* Solander ex Ellis)

Different populations used carnivorous plants for hundreds of years in traditional medicine all around the World. In Europe and North America, butterworts (*Pinguicula vulgaris, Pinguicula alpina*) were used for the treatment of wounds. Decoctions of butterworts and sundew (*Drosera rotundifolia*) were administered for their expectorant and antitussive properties to people with respiratory diseases like pertussis, bronchitis, and asthma but also to treat stomach pain and tuberculosis. Magic properties of sundews were also used for their aphrodisiac effects and their ability to promote delivery. Today this type of plant is used by the modern pharmaceutical industry in the preparation of syrups to treat coughs. The fresh juice secreted by the leaves of sundew is used for local application on warts or bunions (35–38).

In North America, roots and leaves of the purple pitcher plant *Sarracenia purpurea* were used by the endogenous population for its diuretic and laxative properties and also to treat fever, cough, and diabetes. The plant was also used to treat other infectious diseases like scarlet fever, smallpox, and measles. Plant decoctions were also prescribed to pregnant women to ease labor, to prevent sickness after childbirth and to treat absence of menstrual cycle (35–37, 39, 40).

In South-East Asia and in India, natives from local tribes used the pitcher plant *Nepenthes khasiana* as medical plant. They used juice of young flowers and of unopened pitchers or crushed pitcher powder to treat stomach pain and eye troubles (pain, cataract, night blindness), urinary troubles but also skin diseases. Preparations were also given to malaria, leprosy, and cholera patients (41–46).

**Figure 1:**
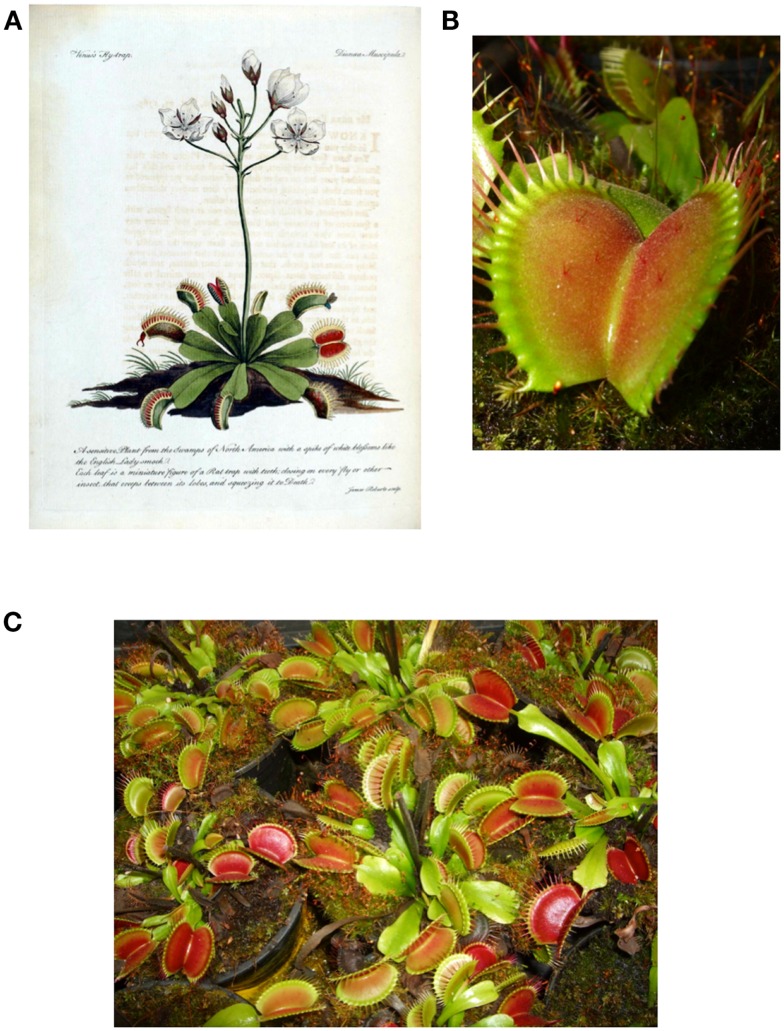
**Picture and illustration of *Dionaea muscipula* Solander ex Ellis**. **(A)** First drawing of Venus flytrap from the first botanical description made by John Ellis and send to Carl von Linné around 1770. **(B)** Picture of *Dionaea muscipula* trap, sensitive hairs are located on the inner face of the trap. **(C)** Picture of *D. muscipula* cultivated by the first author in his own greenhouse.

The Venus flytrap (*D. muscipula* Solander ex Ellis), the only species of the genus *Dionaea*, is a carnivorous plant that grows in marshy areas of North and South Carolina states of the United States (Figure 1). To survive in these environments that are poor in nutrients, it has developed active traps to catch small prey (insects, spiders) that serve as an additional source of nutrients. The plant catches its prey with nectar produced by glands localized at the inner side of the trap and exposing an UV pattern. When the animal touches a sensitive trigger hair, a movement of ions is generated, producing an osmotic gradient that changes the size and shape of specialized cells of the trap that result in trap closure (47–49). Once the trap closes on the prey, other glands, also localized at the inner part of the trap, secrete a digestive acid liquid containing a number of enzymes (proteases, nucleases, phosphatases, and amylases) for digestion of the prey (50). Nutrients are released and then reabsorbed by the plant through both digestive glands and by endocytosis (51–53).

### Secondary metabolites of *Dionaea muscipula*

**Figure 2:**
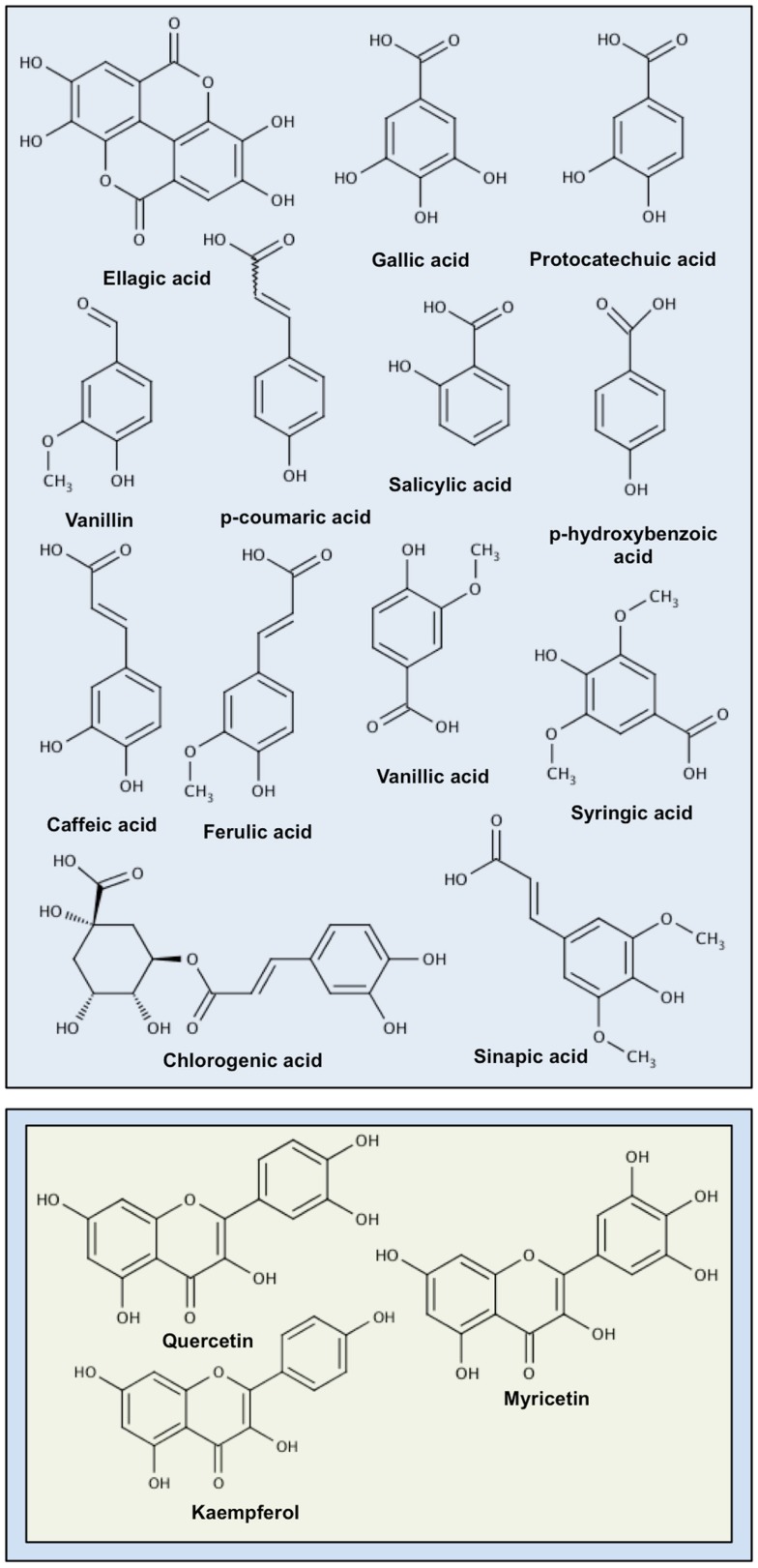

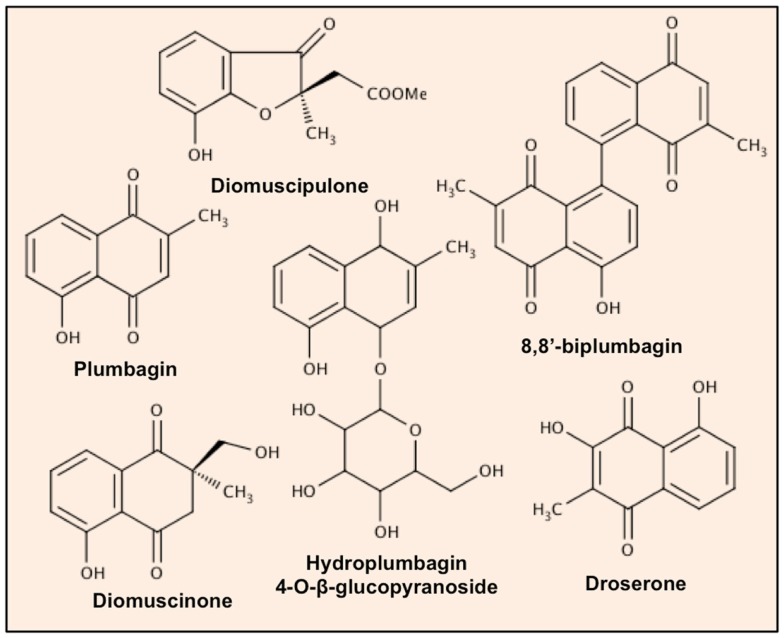
**Chemical structures of molecules present in *Dionaea muscipula* Solander ex Ellis**. Compounds are arranged according to the following classification: phenolic compounds in blue, flavonoids in green, and naphthoquinones in orange.

*Dionaea muscipula* was also the subject of modern biomedical research. The analysis of the various different secondary metabolites (naphthoquinones, flavonoids, phenolic acids) isolated from the plant and identified (Table 1; Figure 2) revealed that they possess different interesting therapeutic properties (54–58).

**Table 1:** **Listing of molecules present in *Dionaea muscipula* Solander ex Ellis**.

Chemical class	Compound	Source other than *Dionaea muscipula*	Biological properties	Reference
Naphthoquinones	Plumbagin	*Drosera* sp., *Juglans* sp., *Limonium axillare, Nepenthes gracilis, Nepenthes khasiana, Plumbago zeylanica*	Anti-bacterial, anti-fungal, anti-parasitical agent, ROS generator, apoptotic agent, cell cycle blocker, Akt, NF-κB pathway inhibitor, Akt, JNK and p38 pathway activator, angiogenesis inhibitor, microtubule inhibitor	Hsieh et al. (59), Bashir et al. (60), Binder et al. (61), Hedin et al. (62), Raj et al. (63), Aung et al. (64), Kawiak et al. (65), Acharya et al. (66), Hsu et al. (67), Sandur et al. (68), Li et al. (69)
	8,8′-biplumbagin (maritinone)	*Diospyros maritima, Plumbago zeylanica*	Anti-microbial agent, cytotoxic agent	Pakulski and Budzianowski (57 ), Gu et al. (70 ), Lin et al. (71 ), Whitson et al. (72 )
	3-chloroplumbagin	*Drosophyllum lusitanicum, Plumbago zeylanica*	n.d.	Kreher et al. (14), Budzianowski et al. (73), Bringmann et al. (74), Sidhu and Sankaram (75)
	Droserone	*Drosera peltata, Nepenthes* sp., *Triphyophyllum peltatum*	n.d.	Kreher et al. (14), Raj et al. (63), Likhitwitayawuid et al. (76), Li et al. (69)
	Diomuscinone	*Diospyros wallichii*	n.d.	Miyoshi et al. (77), Salae et al. (78)
	Diomuscipulone	n.d.	n.d.	Miyoshi et al. (77)
	Hydroplumbagin 4-*O*-β-glucopyranoside	*Drosophyllum lusitanicum*	n.d.	Kreher et al. (14 ), Budzianowski et al. (73 )
Phenolic acids	Ellagic acid, 3-*O*-methylellagic acid, 3,3-di-*O*-methylellagic acid, 3,3-di-*O*-methylellagic acid 4-*O*-glucoside, 3,3-di-*O*-methylellagic acid 4,4′-di-*O*-glucoside, 1-*O*-galloyl-β-glucose	*Drosera peltata, Juglans nigra, Punica granatum, Terminalia chebula* (fruit), Berries, *Vitis rotundifolia*	Apoptotic agent, MAPK, PI3/Akt, NF-κB pathway inhibitor, angiogenesis inhibitor, ABC transporter inhibitor	Pakulski and Budzianowski (57), Aguilera-Carbo et al. (79), Cardona et al. (80), Huang et al. (81), Pellati et al. (82), Edderkaoui et al. (83), Malik et al. (84), Li et al. (69), Tan et al. (85)
	Gallic acid	*Terminalia chebula, Citrus aurantium, Rhodomyrtus tomentosa, Rubus niveus, Vitis* sp., Carrot	Apoptotic agent, Anti-inflammatory agent, cell cycle blocker, kinase inhibitor	Kovacik et al. (54), Pellati et al. (82), Karimi et al. (86), Lai et al. (87), Sultana et al. (88), Verma et al. (89), Weidner (90) (#120), Chandramohan Reddy et al. (91), León-González et al. (92)
	Vanillin	Small fruit seeds, potato, *Ficus microcarpa, Vanilla planifolia*	Growth inhibitor, apoptotic agent, matrix metalloproteinase inhibitor, PI3/Akt, NF-κB pathway inhibitor, angiogenesis inhibitor	Kovacik et al. (54), Ao et al. (93), Cottle and Kolattukudy (94), Lirdprapamongkol et al. (95), Lirdprapamongkol et al. (96), Lirdprapamongkol et al. (97), Shahidi and Perera (98)
	Vanillic acid	Brown rice, small fruit seeds	Cytotoxic agent	Kovacik et al. (54), Hudson et al. (99), Shahidi and Perera (98)
	Protocatechuic acid	*Alpinia oxyphylla, Hibiscus sabdariffa, Rhizoma homalomena, Spatholobus suberectus*	Apoptotic agent, NF-κB pathway inhibitor, Matrix metalloproteinase inhibitor	Kovacik et al. (54), Chen et al. (100), Lin et al. (101), Qing et al. (102), Tang et al. (103), Lin et al. (104), Anter et al. (105)
	Caffeic acid	*Vitis* sp., *Bellis perennis*, coffee beans, *Punica granatum, Hyssopus officinalis*	ROS generator, apoptotic agent, Anti-inflammatory agent, NF-κB pathway inhibitor, Cell cycle blocker	Kovacik et al. (54), Weidner et al. (90), Scognamiglio et al. (106), Rajendra Prasad et al. (107), Moon et al. (108), Jaganathan (109)
	Chlorogenic acid	Coffee beans, *Prunus domestica, Lonicera japonica*	Genotoxic agent, ROS generator, apoptotic agent	Kovacik et al. (54), Bouayed et al. (110), Moores et al. (111), Zhang et al. (112), YANG et al. (113)
	Ferulic acid	Brown rice, small fruit seeds, pineapple, *Vitis* sp.	Anti-oxidant agent	Kovacik et al. (54), Weidner et al. (90), Hudson et al. (99), Shahidi and Perera (98), Graf (114)
	Salicylic acid	*Salix* sp.	Anti-metabolism agent, anti-inflammatory agent, apoptotic agent	Kovacik et al. (54), Hayat et al. (115), Zitta et al. (116), Spitz et al. (117)
	Syringic acid	*Tamarix aucheriana*, white sorghum	Cell cycle blocker, apoptotic agent, angiogenesis inhibitor, NF-κB pathway inhibitor, Proteasome inhibitor	Kovacik et al. (54), Afify Ael et al. (118), Abaza et al. (119)
	*p*-hydroxybenzoic acid	White sorghum, carrot	n.d.	Kovacik et al. (54), Afify Ael et al. (118), León-González et al. (92)
	Sinapic acid	Brown rice, small fruit seeds	Proliferation inhibitor, ABC transporter inhibitor	Kovacik et al. (54), Kampa et al. (120), Hudson et al. (99), Shahidi and Perera (98)
	*p*-coumaric acid	Brown rice, small fruit seeds, white sorghum	Proliferation inhibitory	Kovacik et al. (54), Hudson et al. (99), Shahidi and Perera (98), Tan et al. (85)
Flavonoids	Quercetin, Quercetin 3-*O*-glucoside, Quercetin 3-*O*-(2″-*O*-galloylglucoside), Quercetin 3-*O*-galactoside, Quercetin 3-*O*-(2″-*O*-galloyl)galactoside	*Drosera peltata, Ginkgo biloba, Nepenthes gracilis, Sarracenia purpurea*,	ROS generator, Cell cycle blocker, NF-κB, Wnt pathway inhibitor, Apoptotic agent, kinase inhibitor	Aung et al. (64), Muhammad et al. (121), Park et al. (122), Shan et al. (123), Vidya Priyadarsini et al. (124), Bishayee et al. (125), Kang et al. (126), Li et al. (69), Pakulski and Budzianowski (127)
	Myricetin	*Chamaecyparis obtusa, Jatropha curcas*, Berries	Genotoxic agent, Cell cycle blocker, Apoptotic agent, Akt pathway inhibitor, Matrix metalloproteinase inhibitor	Hakkinen et al. (128), Oskoueian et al. (129), Zwolak et al. (130), Sun et al. (131)
	Kaempferol, kaempferol 3-*O*-galactoside, kaempferol 3-*O*-glucoside	*Drosera peltata, Ginkgo biloba, Gynura medica, Nepenthes gracilis, Pteridium aquilinum*	Apoptotic agent, angiogenesis inhibitor, topoisomerase inhibitor, proteasome inhibitor	Aung et al. (64), Calderon-Montano et al. (132), Kang et al. (126), Luo et al. (133), Luo et al. (134), Li et al. (69), Pakulski and Budzianowski (127)

*n.d., non-defined*.

### Naphthoquinones

These pigment molecules are widespread in plants, lichens, fungi, and microorganisms and these molecules derive from the phenol synthesis pathway. In plants, they act as bactericide, insecticide, fungicide, and allelopathic agents (substances that promote or impede the growth of surrounding organisms) (70, 135, 136).

#### Plumbagin

Plumbagin (Figure 2) is a yellow naphthoquinone with anti-bacterial, anti-fungal, anti-inflammatory, and anti-cancer properties. This molecule gets its name from the plant in which it was discovered, *Plumbago zeylanica* (59) but is very common and is present in others plants like *Limonium axillare* or walnut trees (*Juglans* sp.) (60–63) but also in other carnivorous plants like *N. khasiana, Nepenthes gracilis*, or *Drosera binata* (64, 137, 138). The roots of *P. zeylanica* were already used for centuries in Indian traditional medicine for their cardiotonic, neuroprotective, and hepatoprotective properties (139). In the Venus flytrap, plumbagin provides a protective role against predators and parasites (58).

Capable to generate reactive oxygen species (ROS) and to induce DNA cleavage, plumbagin inhibits topoisomerase II in HL-60 cells (65). It also has a cytotoxic effect on A549 cells and is described as being able to disrupt the microtubular network by interacting directly with tubulin (66). This particular naphthoquinone is known to be an inhibitor of the activated NF-κB (Nuclear Factor kappa B) signaling pathway induced by carcinogens, inflammatory stimuli and TNF-α (Tumor Necrosis Factor alpha). It blocks the expression of anti-apoptotic genes including Bcl-2, Bcl-xL, and surviving and genes regulating cell proliferation (cyclin D1) and angiogenesis like Matrix metalloproteinase 9 (MMP-9) or Vascular endothelial growth factor (VEGF). It thus leads to cell cycle arrest at the G2/M phase transition and an increase of the TNF-induced apoptosis (67, 68). Described as an Akt pathway inhibitor, it also blocks the activity of GSK-3β (Glycogensynthase kinase 3 beta) protein kinase in human breast cancer cell lines MCF-7 and MDA-MB-231 (140). In human melanoma A375.S2 cells, it induces cell cycle arrest in G2/M, which leads to apoptosis. The mechanistic analysis showed that plumbagin activates JNK (c-jun-N-terminal kinase) and ERK (Extracellular signal-regulated protein kinase) 1/2 but had no effect on p38 (141). In H460 lung cancer cells, plumbagin increases the expression of p53 and p21, which leads to cell cycle arrest in G2/M and triggers death by apoptosis. In addition, the authors showed that naphthoquinone activates both JNK and p38 but at the same time inhibits the activity of Akt (142). However in another study, plumbagin has been shown to activate both Akt and ERK 1/2 in healthy pre-adipocyte 3T3-L1 mouse cells (143). *In vivo* experiments performed on mice have shown that plumbagin inhibits the growth of tumors and the number of metastasis by an inhibition of the expression of several markers like MMP-9, 2, and VEGF in ovarian and prostate-cancer cells (144, 145). Due to its structure, plumbagin is also known as a ROS generator. In MCF-7 cells, increased ROS accompanies a decrease of cell viability. Analysis of the mechanism triggered by ROS suggests that plumbagin inhibits 1, 4-phosphatidylinositol 5-kinase (PI5K) expression. In K562 cells, naphthoquinone up-regulates the membrane level of death receptors (DRs) DR4 and DR5, which results in a higher sensitivity to TRAIL (TNF-related apoptosis-inducing ligand) and a reduction of cell viability. Results obtained by molecular docking showed that plumbagin docks into the receptor ligand site of Tumor Necrosis Factor-Related Apoptosis-Inducing Ligand (TRAIL)-DR 5 complex that contributes to explain triggering of apoptosis *via* the extrinsic pathway (146–149). Plumbagin is also known to act as an inhibitor of multidrug resistance-linked ATP-binding cassette drug transporter ABCG2, a protein responsible for the drug efflux in cancer cells (150). *Ex vivo* and *in vitro* experiments showed that plumbagin inhibits microtubule polymerization by direct binding to tubulin at the colchicine binding site (66). Pharmacokinetic studies have shown that plumbagin has an oral bioavailability of about 40% in conscious freely moving rat models and that plumbagin is detected in a micromolar range 1 h after administration (151).

#### Plumbagin derivatives

Some others plumbagin derived molecules have also been isolated from *D. muscipula* by several groups. A plumbagin-dimer, 8,8′-biplumbagin also called maritinone (Figure 2) have also been isolated from the carnivorous plant (57) but also from other plants like *P. zeylanica* or Malaysian persimmon (*Diospyros maritima*) (71, 72). Tested for its potent anti-tumoral effect on KB, LNCaP, Lu1, K562, Raji, Jurkat, Vero, Calu-1, HeLa, and Wish cancer cell lines, maritinone has shown strong effects on the proliferation of these cells (70, 71). Identified in other plants than *D. muscipula* like Malaysian persimmon (*Diospyros maritime*) and carnivorous plants *Nepenthes* sp., and *Drosophyllum lusitanicum* (73, 76, 137, 152) and isolated by Kreher et al. droserone (Figure 2), 3-chloroplumbagin (Figure 2), and hydroplumbagin 4-*O*-β-glucopyranoside (Figure 2) (14) have not yet been studied for their biological effects. Miyoshi et al. reported the isolation of two other naphthoquinones, diomuscinone (Figure 2), and diomuscipulone (Figure 2) together with plumbagin from roots of Venus flytrap (77). Recently, diomuscinone has been isolated from *Diospyros wallichii* (78) but none of these three compounds have been tested to elucidate their biological effects out of the plant.

### Phenolic acids

The term “phenolic” or “polyphenol” is chemically defined as a molecule, which possesses at least one aromatic ring (phenol) or several (polyphenol) hydroxyl substituents. They have many roles in plants like UV sunscreens, messengers, pigments, plant growth factors and protection against fungi, bacteria, insects, and nematodes (153).

#### Ellagic acid

Ellagic acid (Figure 2) is a polyphenolic molecule synthesized by Venus flytrap and many other plants such as pomegranate (*Punica granatum*), *Terminalia chebula* fruit (yellow myrobalan), berry fruits (blueberry, blackberry, and strawberry), *Vitis rotundifolia* (Muscadine grapevine), or black walnut (*Juglans nigra*) (57, 79–82).

By their astringent taste, ellagic acid, and other tannins play a role in plant defense against herbivores and pests as digestibility-reducing compounds (154, 155) but also as anti-bacterial agent (156, 157).

It is a chemopreventive agent that reduces cell proliferation, inhibits NF-κB by interfering with the binding of this transcription factor to DNA. The compound triggers apoptosis of pancreatic cancer cells by cytochrome *c* release and activation of caspase-3 (83). Ellagic acid decreases human prostate carcinoma PC3 cells cell growth and viability in a dose-dependent manner and triggers apoptosis. Authors observed poly(ADP-ribose) polymerase (PARP)-1 cleavage, decrease of anti-apoptotic Bcl-2 protein and increase of pro-apoptotic Bax protein and activation of caspase-3, 6, 8, and 9. Pre-treatment with pan-caspase inhibitor (Z-VAD-FMK) has confirmed the caspase-dependent apoptosis induced by ellagic acid (84). *In vivo* experiments performed on rat models with inducible colon cancer have shown that ellagic acid reduces expression of NF-κB, COX-2 (Cyclooxygenase-2), iNOS (inducible nitric oxide synthase), TNF-α, and IL-6 (158). Using human breast cancer MDA-MB-231 cells and human umbilical vein endothelial cells (HUVEC), Wang et al. have shown that ellagic acid inhibits proliferation, migration, and endothelial cell tube formation. Inhibiting VEGFR-2 tyrosine kinase activity and the downstream signaling pathways including MAPK (Mitogen-activated protein kinase) and PI3K (Phosphatidylinositide 3-kinases)/Akt, ellagic acid decreases MDA-MB-231 breast cancer xenograft growth and p-VEGFR-2 expression. Further *in silico* molecular docking simulations showed that ellagic acid could bind within the ATP-binding region of the VEGFR-2 kinase unit (159).

#### Gallic acid

Gallic acid (Figure 2) has been isolated from bitter orange tree flowers (*Citrus aurantium*), *Marrubium persicum*, yellow myrobalan fruit (*T. chebula*), *Acalypha australis, Pleurotus* sp., *Vitis* sp. seeds, rose myrtle (*Rhodomyrtus tomentosa*), Mysore raspberry (*Rubus niveus*), white sorghum, or carrot (82, 86–88, 90, 118, 160–162).

This tannin that can be released by the aerial parts of the plant is a nematicide but possesses also anti-bacterial and anti-fungal properties (88, 163, 164).

Described in many papers as an anti-cancer agent that can affect many cellular targets (89), gallic acid induces cell cycle arrest in G0/G1 in human leukemia K562 cells by down-regulating cyclin D and E levels. Gallic acid induces cell death by apoptosis in K562 leading to PARP-1 cleavage, cytochrome *c* release, and caspase activation. Expression of COX-2, a molecule involved in cancer-related inflammation and progression, is also reduced by gallic acid treatment. Furthermore, this phenolic acid inhibits BCR/ABL tyrosine kinase and NF-κB pathway activity (91). Moreover, this vegetable tannin blocks Akt/small GTPase and NF-κB pathway activity in human gastric carcinoma AGS cell line and inhibits cellular migration *via* the expression of RhoB. Results have been confirmed in nude mice models where gallic acid treatment leads to decreased development of metastasis (165). *In vivo* experiments using a mouse prostate TRAMP model fed with gallic acid showed inhibition of prostate-cancer growth and progression. Western-blot analysis performed on mice prostate tissues revealed decreased cdc2, Cdk2, Cdk4, and Cdk6 expression as well as a reduction of the proteins cyclin B1 and E (166). Two human osteosarcoma cell lines U-2OS and MNNG/HOS treated with gallic acid allowed demonstrating inhibition of cell proliferation and induction of apoptotic cell death. Results show that gallic acid increases p38 and ERK 1/2 activation and decreases of JNK. Moreover, pre-treatment with a p38 inhibitor prevents gallic acid-induced growth inhibition but not ERK 1/2 and JNK inhibitors that promotes proliferation. Inhibition of tumor growth is confirmed by *in vivo* experiments in a dose-dependent manner in nude BALB/c mice. Immunohistochemistry shows a decrease of PCNA (Proliferating Cell Nuclear Antigen) and CD31 expression in MNNG/HOS tumor tissues (167). Pharmacokinetic studies have shown that gallic acid is rapidly absorbed by the organism, metabolized in different forms after 2 h, and are detected at a micromolar range in plasma, a concentration lower than the concentration used for several biological studies. A study conducted on black tea drinkers showed that after 3 h, the organism eliminates nine different metabolized forms of gallic acid *via* the urinary tract (168, 169).

#### Vanillin

Vanillin (Figure 2) is probably one of the most famous flavor molecules and the most used widely used by food, chemical and perfume industries. Isolated in 1858 by Gobley as the main flavor constituent of vanilla (*Vanilla planifolia*), but also present in other plants (potatoes, *Ficus microcarpa*) (83, 93, 94), vanillin is today mainly synthesized or produced by chemical or biotechnological methods using fungi or bacteria (170–173). In addition to being a flavor molecule, vanillin exerts anti-fungal, and anti-bacterial properties (174, 175).

At non-toxic concentrations, vanillin inhibits growth of mammary adenocarcinoma cell line 4T1 but also decreases MMP-9 activity and thus reduces cell migration and invasion. *In vivo* experiments performed on 4T1 mammary adenocarcinoma cells injected in BALB/c mice have shown that vanillin strongly reduces the number of lung metastasis colonies. Similar experiments performed with vanillic acid were not conclusive (95). Further experiments performed by the same group have shown that vanillin pre-treatment of Hela cells blocks TRAIL – induced phosphorylation of subunit p65 and transcriptional activity of NF-κB pathway and stimulates TRAIL-induced cell death through the extrinsic apoptosis pathway (96). Vanillin also inhibits cell migration of human lung cancer cells induced by hepatocyte growth factor (HGF). It prevents Akt phosphorylation but has no effect on Met and ERK phosphorylation and inhibits phosphatidylinositol 3-kinase (PI3K). Chick chorioallantoic membrane assays showed that vanillin inhibits also angiogenesis (97). Vanillin induces apoptosis in HT-29 human colorectal cancer cell line and NIH/3T3 normal cell lines with a concentration of 400 and 1000 μg/mL, respectively. Flow cytometry analysis showed that a low concentration of vanillin induce cell cycle arrest in G0/G1 phase whereas a high concentration stops cells in G2/M phase (176). Pharmacokinetic studies on rat models demonstrated that vanillin has a relatively good bioavailability (7.6%). Others studies have revealed that 24 h after ingestion, vanillin is mainly metabolized as glucuronide and sulfate conjugates and that after 48 h, 94% of the initial dose of vanillin is found under different forms, including vanillin itself (7%) (177, 178).

#### Protocatechuic acid

Described by many articles as therapeutic molecules active against several diseases, protocatechuic acid (Figure 2) was identified in plants like True roselle (*Hibiscus sabdariffa*), *Rhizoma homalomenae, Spatholobus suberectus*, and *Alpinia oxyphylla* (100–103, 179).

Protocatechuic acid inhibits AGS (human stomach adenocarcinoma) cell migration and proliferation at non-toxic concentrations. It can also inhibit the NF-κB pathway and both MMP-2 expression and activity by modulating RhoB/protein kinase Cε (PKCε) and Ras/Akt cascade pathways. Using *in vivo* mice models (B16/F10 melanoma cells), anti-metastasis proliferation of protocatechuic acid has been confirmed (104). Phenolic acid induces cell death of HepG2 hepatocellular carcinoma cells and stimulates c-Jun N-terminal kinase (JNK) and p38. Further experiments have shown that pre-treatment of HepG2 with *N*-acetyl-l-cysteine (NAC) blocks the cytotoxic effect of protocatechuic acid (180). Protocatechuic acid doesn’t exert genotoxic effects toward *Drosophila melanogaster* wing spot assay. However it shows antigenotoxic effects against hydrogen peroxide inhibits tumoricidal activity and moreover triggers cell death by apoptosis in HL-60 leukemia cells (105).

#### Caffeic acid

Present in *Vitis* sp. seeds, pomegranate (*P. granatum*), coffee beans, honey, common daisy (*Bellis perennis*), and hyssop (*Hyssopus officinalis*), caffeic acid (Figure 2) is a secondary metabolite that exerts anti-bacterial and anti-fungal properties (90, 106, 181, 182).

Caffeic acid is a ROS generator inducing oxidative DNA damage and alters mitochondrial membrane potential in HT-1080 human fibrosarcoma cells. It stimulates lipid peroxidation and decreases activities of enzymatic anti-oxidants superoxide dismutase (SOD), catalase (CAT), as well as glutathione peroxidase (GPx), and glutathione (GSH) levels. Observations by fluorescence microscopy showed that caffeic acid induces cell death by apoptosis (107). This molecule is known to act as an inhibitor of DNA methylation due to its ability to inhibit human DNA methyltransferase 1 (DNMT1) and to partially inhibit retinoic acid receptor (RAR) b promoter in MCF-7 and MAD-MB-231 cells (183). Caffeic acid is an anti-inflammatory agent by decreasing expression of IL-8 and NF-κB pathway activity by triggering TNF-alpha-induced IκB degradation that lead to a reduction of NF-κB target genes expression which are regularly involved into carcinogenesis (108). Caffeic acid decreases HCT 15 colon cancer cells in a time dependent manner. It induces cell cycle arrest that leads to accumulation of cells in sub-G1. Inducing also ROS production and reduction of the mitochondrial membrane potential, flow cytometry analysis confirmed cell death by apoptosis (109). Among several small phenolic acids tested for their anti-proliferative effect on T47D human breast cancer cells, caffeic acid exerts is most potent. Further experiments showed that all compounds induce apoptosis *via* the Fas/FasL pathway and that caffeic acid is able to inhibit aryl hydrocarbon receptor-induced *CYP1A1* gene expression (120). However it is important to underline that chlorogenic acid (Figure 2), a caffeic acid analog and a Venus flytrap secondary metabolite, can be hydrolyzed to caffeic acid in the intestine and can be well absorbed by intestinal cells. *In vitro* and *in vivo* studies showed that in Caco-2 cells, caffeic acid exerts stronger anti-oxidant properties compared to chlorogenic acid. This differential efficiency can be explained by the fact that caffeic acid uptake is superior to chlorogenic acid uptake. Caffeic acid is a molecule known to be metabolized by intestinal bacteria, however studies have shown that caffeic acid can be detected in rat blood 6 h after ingestion together with different other metabolites. Another study demonstrated that 95% of caffeic acid is absorbed and that 11% of the ingested caffeic acid was excreted in urine (182, 184–187).

#### Chlorogenic acid

Chlorogenic acid (Figure 2) has been isolated from a huge diversity of plants like prune (*Prunus domestica*), japanese honeysuckle (*Lonicera japonica*), apple, plum, *Eucommia ulmodies*, and coffee beans (110–112, 188–190).

In plants, chlorogenic acid is a secondary metabolite involved in plant defense against pests, herbivores, fungi, or virus (191–194).

Human adenocarcinoma Caco-2 cells treated with chlorogenic acid present a reduced proliferation rate and light microscopy observation reveals an abnormal morphology compared to untreated cells (195). Chlorogenic acid induces apoptosis by inducing ROS generation and reduces the mitochondrial membrane potential in U937 human leukemia cells. Further results obtained by Western-Blot show that chlorogenic acid promotes caspase-3 activity and expression of caspase-3, 7, 8, and 9 in U937 cells (113). Chlorogenic acid can induce DNA damage in both normal lung MRC5 fibroblasts and A549 lung cancer cells and increases the levels of topoisomerase I- and topoisomerase II-DNA complexes in cells although cancer cells were the most sensitive to chlorogenic acid treatment (196). This compound also acts as an anti-oxidant reducing free radical DNA damages like DNA-single strand breaks (110, 197).

Chlorogenic acid has a very low bioavailability but is always present in the small intestine. It can only be detected in rat plasma with other metabolites in trace amounts 6 h after absorption. Another study has given the same result, chlorogenic acid has a rate of absorption of 33% and is detected only in trace amounts in rat urine. Studies showed that chlorogenic acid is not well absorbed by the organism compared to structurally related caffeic acid. Caffeic acid metabolism produces caffeic and ferulic acid, two other secondary metabolites of *D. muscipula* (184–186).

#### Ferulic acid

Ferulic acid (Figure 2) has been identified mainly in seeds like *Vitis* sp. seeds brown rice, but also wheat flour, pineapple, creosote bush (*Larrea divaricata*). Ferulic acid is an allelopathic agent that acts as seed germination inhibitor (98, 99, 90, 198–202, 222).

Ferulic acid pre-treatment protects against γ-radiation-induced DNA damage in hepatocytes and significantly increases anti-oxidant enzymes, GSH, vitamins A, E, and C (203). *In vivo* studies have shown that mammary carcinogenesis induced in Sprague–Dawley rats fed with ferulic acid prevent tumor development in 80% of animals even if the exact protection mechanism remains unclear (204). Further animal experimentations on induced skin carcinogenesis mice model highlighted that oral ferulic acid administration completely prevented skin tumor formation but that topical application does not (205). Ferulic acid delayed cell cycle progression of Caco-2 colon cancer cells. cDNA microarrays showed that ferulic acid up-regulates centrosome assembly genes, such as *RABGAP1* and *CEP2* and S phase checkpoint protein *SMC1L1* (206). Moreover, ferulic acid acts as an anti-oxidant that can reduce DNA strand breaks induced by γ-irradiation in peripheral blood leukocytes and bone marrow cells of mice. It promotes mice survival up to 6 Gy of γ-radiation (114, 207). Ferulic acid is absorbed by the intestine and can be detected in the blood of rat and human patients. Further studies showed that ferulic acid can be absorbed very quickly all along the gastrointestinal tract, can be detected in plasma already after 10 min and less than 1% of ingested ferulic acid can be found in rat feces. It can be metabolized under different forms including glucuronides, sulfates, and sulfoglucuronides conjugated forms, formed in the liver by different phase II enzymes reduce bioavailability (202, 208).

#### Salicylic acid

Already used by the Greeks and the Egyptians to treat aches and pains, this compound was initially isolated from willow tree bark by Buchner (Figure 2) in 1898. The isolated active principle was named from the Latin word “*Salix*” which means willow tree. Salicylic acid has been identified as the main metabolite of acetylsalicylic acid, the active principle of aspirin. Salicylic acid is a phytohormone that plays important roles in plant immune system, thermogenesis (heat production), root nodule formation but also more essential process like metabolism, flowering, and seed germination. Due to its important role, salicylic acid is found in almost all plants (115, 209–213).

Salicylic acid has no effect on CaCo-2 (colon carcinoma cells) proliferation under normoxic conditions but increases caspase-3/7 activities and increases phosphorylation of ERK 1/2 under hypoxic conditions: salicylic acid increases caspase-3/7 activities but also decreases cell proliferation but has no effect on ERK 1/2 phosphorylation (116). *In vitro* assays have shown that salicylic acid reversibly inhibits 6-phosphofructo-1-kinase, an enzyme responsible for the glycolysis. It dissociates the quaternary structure of the enzyme into inactive dimers. Tested on MCF-7 cells, salicylic acid inhibits 6-phosphofructo-1-kinase that leads to a decreased cellular glucose consumption and viability (117). Anacardic acid, a derivative of salicylic acid and an inhibitor of histone acetyltransferase, is an anti-inflammatory compound like its precursor. It blocks the NF-κB pathway by abrogation of phosphorylation and degradation of IκBα and by inhibiting acetylation and nuclear translocation of its p65 subunit. Inhibition of the NF-κB pathway leads to down-regulation of target genes involved in cell proliferation (cyclin D1, COX-2), survival (Bcl-2, Bcl-xL), and invasion (MMP-9) (213, 214). Several clinical trials analyzed the effect of salicylic acid on colorectal cancer patients. Results show that a dose of 75 mg of aspirin per day during several years reduces colorectal cancer incidence and mortality (215).

### Flavonoids

Flavonoids are secondary metabolites of the polyphenol family with a backbone composed of 15 carbon atoms organized into a common phenyl benzopyrone structure (C6-C3-C6). This group of molecules is divided into several sub-groups according to their chemical formulations including flavonols (quercetin, myricetin, and kaempferol), flavones, flavanones, flavanols, anthocyanins, dihydroflavonols, isoflavones, and chalcones. Their roles within plants are very diverse. Some have a protective role against UV, but also toward parasites, pathogens (insecticides, fungicides, vermicides) and herbivores. Other molecules act as signal molecules or help the plant to survive under stress conditions (drought period, nutrient-poor environment) (216).

#### Quercetin

Quercetin (Figure 2) is a molecule with anti-bacterial properties present in bitter orange tree flowers (*Citrus aurantium*), *Epilebium* species, *Nepenthes gracilis, Leucaena leucocephala, S. purpurea*, caper (*Capparis spinosa*), and chili peppers (*Capsicum* sp.) (64, 86, 121, 217–221). In plants, quercetin acts as a host defense molecule and a growth stimulatory agent, it is a nematode repellant, an anti-microbial agent, a root nodules inducer, an allelopathic agent, and a hyphal branching attractor for symbiotic fungus (223).

Concerning its biological properties as anti-cancer agent, quercetin has been the object of many studies (224). For example, this flavonol has been described as an anti-proliferative agent by inducing cell cycle arrest in G2/M and as an apoptotic agent due to its ability to inhibit the transcriptional activity of the Wingless pathway (Wnt) by reducing the amount of transcriptional co-activator β-catenin in the nucleus in SW480 colon cancer cells and by reducing the level of cyclin B1 and surviving (122, 123). *In vitro* experiments have shown that a concentration of 2 μM of quercetin is sufficient to decrease 80% of the activity of 16 kinases, which are mostly involved in the control of mitotic processes (225). This secondary metabolite is also responsible for the induction of cell death by apoptosis of hepatocellular carcinoma cells after activation of caspases 3 and 9 (226). Quercetin used in combination with 5-fluorouracil (5-FU) on EC9706 and Eca109 esophageal cancer cells increased the cytotoxic effect and the percentage of apoptotic cells compared to quercetin or 5-iFU alone. These combined effects were explained by a decrease of p-IκBα expression induced by quercetin treatment (227). Quercetin is also known to induce cell cycle arrest in G2/M and to induce cell death in human HeLa cervical cancer cells by mitochondrial apoptosis through a p53-dependent mechanism. These results also showed that quercetin can inhibit the NF-κB pathway by modulating the expression of NF-κB p50 and p65, IKKβ, p-IκB, and ubiquitin. Other results obtained by Western-blot have shown an increase of pro-apoptotic Bcl-2 family proteins (Bax, Bak, and Bad), an up-regulation of Apaf-1 and cytoplasmic cytochrome *c* and a down-regulation of anti-apoptotic Bcl-2 family proteins (Bcl-2, Mcl-1) (124). Moreover Spagnuolo and collaborators demonstrated in addition, in U937 cells, a down-regulation of Mcl-1 by quercetin acting directly or indirectly on its mRNA stability and protein degradation (228). A study performed on HeLa cells showed that quercetin has the ability to interact with DNA and to generate ROS. This flavonol triggers a cell arrest in G2/M, followed by mitochondrial membrane depolarization, externalization of phosphatidyl-serine, release of cytochrome *c* into the cytoplasm, decrease of Akt and Bcl-2 expression and cell death by apoptosis (125). A large Swedish population-based case-control study has shown that quercetin uptake decreases the risk to develop gastric adenocarcinoma. This protective effect was very strong for female smokers (229). Quercetin has been tested in several clinical trials on cancer patients. It has been tested in a chemoprevention purpose on 130 colon cancer patients treated with quercetin, rutin, or with sulindac (NCT00003365). Phase I clinical trials have shown that quercetin inhibits protein tyrosine phosphorylation in patient lymphocytes, is able to decrease CA-125 (Carbohydrate antigen 125) level in patients with ovarian cancer refractory to cisplatin and serum alpha-fetoprotein (AFP) levels in hepatocellular carcinoma patients (230). Quercetin is also actually undergoing clinical trials with genistein to evaluate their effects on prostate-specific antigen level on prostate-cancer patients (NCT01538316). Pharmacokinetic analysis performed on humans and rats have shown that quercetin has a very low bioavailability. In human, after an ingestion of about 87 mg of quercetin, average plasma concentration is 344 nM after 3 h. Results have also shown that quercetin is no longer present in the aglycone, free form but is metabolized, and can only be detected as conjugated derivatives like quercetin glucuronides or quercetin 3-*O*-sulfate. However after further analysis Manach et al. showed that these quercetin derivatives maintain anti-oxidant activity although their effect were reduced to half of the quercetin (231–233). Sesink et al. showed that breast cancer resistance ABCG2 and the multidrug resistance-associated protein 2 (Mrp2), two ATP-binding cassette (ABC) transporters involved in drug cancer resistance are able to pump both quercetin aglycone and quercetin conjugated derivatives out of the cells and thus explain the low bioavailability of quercetin (234).

#### Myricetin

Myricetin (Figure 2) is a quercetin analog present in many plants as for example *Limonium axillare, Jatropha curcas*, Japanese cypress (*Chamaecyparis obtusa), Leucaena leucocephala*, and many berries (60, 128, 129, 218, 235). In plants, myricetin acts as a host defense molecule, is released by roots and acts as a nematode repellent and an inducer of root nodules in several cases (223).

This is a flavonol that exerts anti-bacterial (217) and anti-cancer properties which is able to inhibit mutagenesis induced by carcinogens such as benzo(a)pyrene (236). Myricetin is able to induce apoptosis of pancreatic cancer cells *via* the activation of caspase-3 and 9 (130). It induces apoptosis of human bladder carcinoma cell line T-24 with activation of caspase-3 after DNA cleavage and cell cycle arrest in G2/M phase by a down-regulation of cyclin B1 and cdc2. It inhibits the phosphorylation of Akt but increases the phosphorylation of p38 and decreases MMP-9 expression. *In vivo* experiments have shown a growth inhibition of T-24 xenografts on mice models (131). Myricetin is also able to induce apoptosis in HL-60 (human promyelocytic leukemia cells) through an ROS-independent cell death pathway (237). *In vitro* experiments have shown an inhibition of mammalian DNA polymerases and human DNA topoisomerase II by myricetin. Further experiments have revealed that it also inhibits proliferation of HCT-116 human colon carcinoma cells and trigger apoptosis after a cell cycle arrest in G2/M cell cycle transition (238). Recent studies have shown that a non-toxic dose of myricetin decreases PI3 kinase activity in pancreatic cancers cells MIA PaCa-2, Panc-1, or S2-013 and triggers cell death by apoptosis. *In vivo* experiments performed on mice have shown a regression of tumor growth and a decrease of metastasis (239). In rat models, myricetin is able to inhibit cytochrome P450 (CYP) activity in liver or intestine and thus to increase bioavailability of tamoxifen, a drug used to treat breast cancer. Similar results were observed for doxorubicin (240, 241).

#### Kaempferol

Kaempferol (Figure 2) is a flavonol identified in many plants like *Nepenthes gracilis*, chili peppers, *Gynura medica*, Bracken (*Pteridium aquilinum*), *Ginkgo biloba* (64, 126, 132, 219, 242, 243).

Involved into plant defense, kaempferol has been described as a nematode repellent, nematode egg hatching inhibitor, and allelopathic agent (223).

From a therapeutic point of view, anti-cancer properties of kaempferol have been underlined by many papers (132, 244). A concentration of 40 μM of kaempferol is sufficient to inhibit proliferation of oral cancer cell lines (SCC-1483, SCC-25, and SCC-QLL1). Analysis has shown PARP-cleavage and caspases-3-dependent apoptosis (126). Kaempferol inhibits ovarian cancer cells and induces cell death by apoptosis in a dose-dependent manner. Luo et al. observed caspase-3 and 7 cleavage that was abrogated by caspase 9 inhibitor that confirmed the extrinsic caspase-dependent cell death mechanism. Western-Blot analysis showed an up-regulation of pro-apoptotic proteins Bax and Bad and a down-regulation of anti-apoptotic protein Bcl-xL (133). The same team analyzed effects of kaempferol on VEGF expression in ovarian cancer cells. Results show that this flavonol inhibits time-dependently VEGF secretion and angiogenesis. It also down-regulates phospho-ERK concomitant with c-myc and NF-κB expression through ERK signaling pathway (134). They also developed different kaempferol nanoparticles and have tested their efficiency on cancerous and normal ovarian cells. PEO [poly(ethylene oxide)], PPO [poly(propylene oxide)], PEO poly(ethylene oxide) decreases both ovarian cancer and healthy cell viability. On the opposite (PLGA) [Poly(DL-lactic acid-co-glycolic acid)] exerts selective cytotoxic effect on cancer cells only. However, all kaempferol nanoparticle formulations were able to reduce cancer cell viability better than kaempferol alone (245). Pharmacokinetic *in vivo* studies performed on human and rats have revealed that kaempferol is mainly absorbed in the small intestine and is metabolized to glucurono and sulfo-conjugated forms in the liver. Results have shown that kaempferol has a very poor bioavailability (2%) and that after ingestion of several mg of kaempferol, it is only detected at nanomolar levels in plasma and it should be emphasized that most *in vitro* studies were conducted at micromolar concentrations (132, 231, 246). It has also been showed that kaempferol can be converted into its analog, quercetin (Figure 2) by the enzyme CYP1A1 in rats (247). Although cancer cells are able to eliminate compounds like quercetin, it has been shown by Sesink et al. that kaempferol blocks Bcrp-mediated quercetin efflux by competitive inhibition (234, 248). Based on this discovery, it has been shown that kaempferol enhances the effect of cisplatin in ovarian cancer cells and of etoposide in rat models (249, 250).

## Conclusion

This review has presented the different known chemopreventive and therapeutic agents isolated from *D. muscipula*. At the present time, more than 15 compounds (Figure 2) have been isolated from *D. muscipula*, mostly flavonoids, and phenolic compounds. Most of these secondary metabolites are also present in other plants and up to now, only one *D. muscipula*-specific molecule with therapeutic potential has been isolated from Venus flytrap, diomuscipulone (Figure 2). But this naphthoquinone has not yet been tested for its biological properties like several others compounds as diomuscinone, droserone, 3-chloroplumbagin, and hydroplumbagin 4-*O*-β-glucopyranoside or *p*-coumaric acid (Figure 2) which are also present in other plants. Many of these anti-cancer compounds present in *D. muscipula* have been described as NF-κB pathway modulators like plumbagin, ellagic acid, or salicylic acid. The reason is that the NF-κB pathway is an interesting anti-cancer drug target due to its involvement into the development and the progression of many cancers (251–253). However it’s important to keep in mind that the NF-κB pathway is not responsible for all types of cancer and that there are many other pathways and phenomena involved in cancer development and progression that can be the targets for drugs of natural origins (18, 19, 28, 251, 254, 255).

Currently only several compounds like quercetin, salicylic acid, and kaempferol have moved to pharmacokinetic studies and clinical trials (Table 2). All results have shown that these compounds have a very poor bioavailability that can be explained by several reasons. Plant secondary metabolites are often recognized as xenobiotics by the organism and are rapidly detoxified by gut flora or enzymes and eliminated from the organism. Intestinal bacteria are known to metabolize drugs before their absorption by the organism. Some drugs can be directly metabolized by the organism or can be conjugated and transformed into an inactive molecule before reaching their target. However several studies have shown that it is possible that this defense mechanism can lead to the conversion of an inactive molecule into another one like kaempferol into quercetin. Moreover, it is known that cancer cells use drug resistance mechanisms like ABC transporter efflux pumps to down-regulate intracellular drug levels. It is very important to take theses mechanisms into consideration to understand and develop cancer drugs of natural origins but also all other kinds of drugs. New secondary metabolites generated by the organism during drug metabolism by bacteria, the organism itself, or cancer cells should be identified and taken into consideration. It is also very important to underline that due to the low bioavailability of several of these compounds, they are only present in nanomolar concentration in plasma while in many studies, a concentration in milli or micromolar is used to treat cells in *in vitro* conditions and to obtain an effect. However, it has been shown that co-treatment of two natural molecules like quercetin and kaempferol and a chemotherapeutic drug like cisplatin or etoposide is more efficient than a single treatment thanks to the ability of the natural compound to block ABC transporters. Low bioavailability and incompletely absorbed compounds are ineffective against metastatic and invasive cancers.

**Table 2:** **Clinical trials involving natural compounds present in *Dionaea muscipula* Solander ex Ellis**.

Chemical class	Compound	Trial name	Disease	Status	Identifier
Phenolic acids	Ellagic acid	Dietary intervention in follicular lymphoma	Follicular lymphoma	Unknown	NCT00455416
	Caffeic acid	FLAX FX, A research study of the effects of flaxseed lignans on colon health	Colon cancer	Recruiting	NCT01619020
	Ferulic acid	FLAX FX, A research study of the effects of flaxseed lignans on colon health	Colon cancer	Recruiting	NCT01619020
Flavonoid	Quercetin	Prostate-cancer prevention trial with quercetin and genistein (QUERGEN)	Prostate cancer	Recruiting	NCT01538316
		Sulindac and Plant compounds in preventing colon cancer	Colon cancer	Terminated	NCT00003365

The table was generated by using data available from the website http://clinicaltrials.gov

One of the most promising anti-cancer compounds is probably plumbagin. It has been shown that plumbagin induces cell death, affects many hallmarks of cancer, interacts directly with cancer targets like tubulin, inhibits ABC transporters, is well absorbed by the organism and can be present in the organism at a micromolar concentration. We have to underline that plumbagin is used for centuries in traditional medicines and is present in many plants that can explain that plumbagin is more studied than other Venus flytrap compounds.

Most natural compounds isolated from *D. muscipula* like plumbagin, quercetin, myricetin, ellagic acid, or vanillin have multiple effects and act as anti-cancer drugs with multiple targets on different types of cancers. However, as in many cases, direct drug targets are often unknown and there are several reasons that can explain this situation. Plant secondary metabolites are usually small molecules compared to their protein targets and analytical methods have only been developed recently and this step is usually the most challenging, expensive and the most time consuming in the drug development process. On one hand, the development of new methods, techniques, and devices like high-throughput screening but also of new biological discoveries (new organism, interspecies interactions) will lead to new molecule discovery in already known organisms but also in new species (256–260). On the other hand, discovery of new anti-cancer drug targets, new visions, and new approaches of cancer development by biological experiments as for example identification of immediate drug direct protein and nucleic acid targets by *Drug Affinity Responsive Target Stability* (DARTS) or chemical proteomics (261–263) but also by computational data analysis like molecular docking (264–266) and grouping together all these data in databases like STITCH (Search Tool for Interactions of Chemicals) (267) will also allow investigation of new cancer treatments. Moreover, development and screening of known and new derivatives from Venus flytrap but also from other plants will improve specificity and efficiency of these promising therapeutic compounds (255, 268–272).

Data presented here show that Nature can be considered an impressive medicinal cabinet that remains to be entirely discovered, improved and used by researchers to hit the right targets.

## Conflict of Interest Statement

‘The authors declare that the research was conducted in the absence of any commercial or financial relationships that could be construed as a potential conflict of interest.
